# A Fosmid-Based System for the Generation of Recombinant Cercopithecine Alphaherpesvirus 2 Encoding Reporter Genes

**DOI:** 10.3390/v11111026

**Published:** 2019-11-05

**Authors:** Ekaterina Chukhno, Sabine Gärtner, Abdul Rahman Siregar, Alexander Mehr, Marie Wende, Stoyan Petkov, Jasper Götting, Akshay Dhingra, Thomas Schulz, Stefan Pöhlmann, Michael Winkler

**Affiliations:** 1 Infection Biology Unit, German Primate Center-Leibniz Institute for Primate Research, 37077 Göttingen, Germany; ekaterina.chukhno@gmail.com (E.C.); SGaertner@dpz.eu (S.G.); ASiregar@dpz.eu (A.R.S.); alex.mehr@freenet.de (A.M.); m.wende@tu-braunschweig.de (M.W.); spoehlmann@dpz.eu (S.P.); 2 Faculty of Biology, Universitas Gadjah Mada, Yogyakarta 55281, Indonesia; 3 Platform Degenerative Diseases, German Primate Center-Leibniz Institute for Primate Research, 37077 Göttingen, Germany; SPetkov@dpz.eu; 4 German Center for Cardiovascular Research (DZHK), partner site, 37099 Göttingen, Germany; 5 Institute of Virology, Hannover Medical School, 30625 Hannover, Germany; Goetting.Jasper@mh-hannover.de (J.G.); Dhingra.Akshay@mh-hannover.de (A.D.); Schulz.Thomas@mh-hannover.de (T.S.); 6 Faculty of Biology and Psychology, University Göttingen, 37073 Göttingen, Germany

**Keywords:** Cercopithecine alphaherpesvirus 2, fosmid, recombineering

## Abstract

The transmission of Macacine alphaherpesvirus 1 (McHV-1) from macaques, the natural host, to humans causes encephalitis. In contrast, human infection with Cercopithecine alphaherpesvirus 2 (CeHV-2), a closely related alphaherpesvirus from African vervet monkeys and baboons, has not been reported and it is believed that CeHV-2 is apathogenic in humans. The reasons for the differential neurovirulence of McHV-1 and CeHV-2 have not been explored on a molecular level, in part due to the absence of systems for the production of recombinant viruses. Here, we report the generation of a fosmid-based system for rescue of recombinant CeHV-2. Moreover, we show that, in this system, recombineering can be used to equip CeHV-2 with reporter genes. The recombinant CeHV-2 viruses replicated with the same efficiency as uncloned, wt virus and allowed the identification of cell lines that are highly susceptible to CeHV-2 infection. Collectively, we report a system that allows rescue and genetic modification of CeHV-2 and likely other alphaherpesviruses. This system should aid future analysis of CeHV-2 biology.

## 1. Introduction

Herpesviruses are a large family of DNA viruses that usually establish a lifelong latency or persistence in their hosts. Herpes simplex virus 1 (HSV-1, human alphaherpesvirus 1) and other members of the genus *Simplexvirus*, subfamily *Alphaherpesvirinae*, establish latency in sensory neurons and have a conserved genome structure. Primate simplexviruses coevolved with their host species, resulting in codivergent trees for the viruses and their respective host species [1,2]. Despite this coevolution, these viruses are not species-specific, and cross-species transmission has been frequently observed. The transmission of Macacine alphaherpesvirus 1 (McHV-1, also termed herpes B virus) from macaques to humans causes encephalitis that is associated with a high case fatality rate in the absence of treatment [3]. In contrast, HSV-1 rarely causes encephalitis and the McHV-1-related viruses Cercopithecine alphaherpesvirus 2 (CeHV-2, also termed simian agent 8) and Papiine alphaherpesvirus 2 (PaHV-2, herpes virus papio 2) are believed to be non-pathogenic in humans. However, the molecular determinants of neurovirulence of these primate simplexviruses are largely unknown [4].

CeHV-2 was identified in African vervet monkeys in 1958 [5] and was subsequently found to also infect baboons [6]. Natural infection of these animals is usually asymptomatic. Although, oral and/or genital lesions can be associated with infection [7]. In contrast, experimental infection of vervets (intraspinally) and rabbits (intradermally) was associated with paralysis, and encephalomyelitis, respectively [6]. The genomic sequence of CeHV-2 has been determined [8] and the virus was found to efficiently replicate in a panel of cell lines of different species, including cell lines of human origin, with only dog-derived MDCK cells being largely resistant to infection [9]. The presumed lack of neurovirulence of CeHV-2 in humans suggested that CeHV-2 and McHV-2 might differentially interact with human neuronal cells, or differentially evade control by human immune responses. Investigating these scenarios requires the establishment of experimental systems that allow genetic modification of CeHV-2 and McHV-1.

Two types of E. coli-based recombinant systems for the production of herpesviruses have been described: Cosmids and bacterial artificial chromosomes (BACs). The rescue of herpesviruses from cosmids has been first described for HSV-1 [10] and subsequently for other herpes viruses. However, the introduction of targeted mutations in cosmids is challenging due to their large size [11,12]. Cloning and rescue of large DNA virus genomes into BACs was first reported for baculovirus [13] and mouse cytomegalovirus [14], and was subsequently also applied to HSV-1 [15,16,17]. Moreover, the development of recombination-based methods using phage recombinases (recombineering) allows the modification of large viral genomes in E. coli [18,19,20]. Here, we report cloning of the fragmented CeHV-2 genome into fosmids [21]. This approach has been employed previously for avian herpesviruses and pseudorabies virus [22,23,24,25] and has allowed us to rescue infectious viruses and introduce reporter genes into the viral genome via recombineering. Infection by these recombinant viruses could be readily quantified based on reporter gene expression. The viruses exhibited a broad cell tropism, but were unable to efficiently infect macaque cell lines.

## 2. Materials and Methods

### 2.1. Plasmids and Oligonucleotides

Plasmids for the insertion of the reporter genes into the viral genome, by en passant mutagenesis, contained positive (kanamycin resistance) and negative (I-SceI site) selection markers that were inserted into the reporter gene. These were flanked by a 50 bp duplication of sequences next to the insertion site, as outlined by [18,19]. pcDNA3-EGFP-en was derived by stepwise insertion of EGFP-N-terminus, I-SceI site/kanamycin resistance marker and EGFP-C-terminus into pcDNA3. pmCherry-EP was derived by inserting a PstI fragment from pEP-mRFP1-in containing I-SceI site/kanamycin resistance marker [19] into pRSET-B-mCherry [26]. pcDNA3-iRFP670-en was generated by replacing N- and C-termini of EGFP in pcDNA3-EGFP-en with sequences of iRFP670 amplified from MXS_iRFP670 [27]. To generate pHW2000GG-seg8-A/PR/8/34-M2A-Gluc-en, a fragment containing I-SceI site/kanamycin resistance marker was amplified by PCR, where one primer provided the Gluc sequence duplication, and inserted into the NruI site of the Gluc reporter gene. In a second step, this modified Gluc gene was inserted into pHW2000GG-seg8-A/PR/8/34-M2A-EGFP to generate a P2A-Gluc fusion.

Oligonucleotides were purchased from Sigma-Aldrich (St. Louis, MO, USA). The following oligonucleotides were used for recombineering: ep-SA8-ICP4-Gluc-3: 5′-GGTCGCGGGCGGGGGTCGCGGGCGGGGGTCGCGGGCGGCGGCGCCGGCTAGTCACCACCGGCCCCCTTGATCTT-3′; ep-SA8-ICP4-2A-5: 5′-CGACCCCGGACTGGGACCCGGACGGCGAGCCGGCGGCCGCCGAGGACTGGGGGTCCGGCGGAGCCACAAATTTCTCCCTCCT-3′; ep-SA8-UL10C-R670-5: 5′-ACCACCCCCGGAGGGTCGAGGAGCCCATCTACGAGACCGTGGGCGAGTGGGGGTCCGGCATGGCGCGTAAGGTCGATCTC-3′; ep-SA8-UL10C-R670-3: 5′-CGTCGCGTTAAGAGGCAGTTTGGTTTTTATTGATCGAGGGGCGCGAGTCAGCGTTGGTGGTGGGCGGCGGT-3′; ep-SA8-UL35-EGFP-5: 5′-CCTACCTGCCGTTTCTGGCCGCCCCCGCCGCCCAGCCCGCGCGCAGCCCCGGAGGGTCCGGCATGGTGAGCAAGGGCGAGGA-3′; ep-SA8-UL35-EGFP-3: 5′-ACAGAGGGGGAGGGGGCTGCGGGGCGGGGCGCGCCCGGGAACCGCGCCTACTTGTACAGCTCGTCCATGC-3′; ep-SA8-ICP4-EGFP-5: 5′-CGACCCCGGACTGGGACCCGGACGGCGAGCCGGCGGCCGCCGAGGACTGGGGGTCCGGCATGGTGAGCAAGGGCGAGGAGCTG-3′; ep-SA8-ICP4-EGFP-3: 5′-GGTCGCGGGCGGGGGTCGCGGGCGGGGGTCGCGGGCGGCGGCGCCGGCTACTTGTACAGCTCGTCCATGCCGAG-3′. The following oligonucleotides were used for control of recombineering products: ep-SA8-UL35C-kon5: 5′-CTGGCGCTGCAGCCGATGTT-3′; ep-SA8-UL35C-kon3: 5′-GTCCCACCCCTCAGGCAACA-3′; ep-SA8-UL10C-kon5: 5′-GGGGAGCCCATCTACGACGA-3′; ep-SA8-UL10C-kon3: 5′-CGGGGGGGAAACTCACACGA-3′; ep-SA8-ICP4-kon5: 5′-CTGCTCGCCGACGCCGAGGA-3′; ep-SA8-ICP4-kon3: 5′-CGACACACGATCTGCGAGCTT-3′.

### 2.2. Cell Culture

Vero76, Cos7 (both African green monkey, kidney), cjFF2 (common marmoset *Callithrix jacchus*, fibrobast), A549 (human, lung), LLC-MK2, MaMuK8639 (both rhesus macaque *Macaca mulatta*, kidney, kidney), sMAGI (rhesus macaque *Macaca mulatta*, mammary tumor), TeloRF (rhesus macaque *Macaca mulatta*, fibroblast), and LR-7 cells (mouse) were cultivated in DMEM supplemented with 10% fetal calf serum, 100 U/mL penicillin and 100 μg/mL streptomycin.

The cjFF2 cells were derived using marmoset (*Callithrix jacchus*) foetal material (pregnancy day 70) leftovers from other unrelated, fully approved studies (LAVES license numbers 42502-04-16/2129 and 42502-04-16/2130). Tissue fragments from the dorsal wall and limb buds were digested with a mixture of 1:1 (*v*:*v*) Accutase (Gibco, Carlsbad, CA, USA) and Collagenase IV (Worthington Biochemical Corporation) (2 mg/mL) at 37 °C for 15 min, disaggregated by pipetting, and cultured in gelatin-coated culture dishes with M15 culture medium (Dulbecco’s DMEM containing GlutaMAX (Gibco, Carlsbad, CA, USA) supplemented with 15% FBS (Gibco, Carlsbad, CA, USA), non-essential amino acids (NEAA (Gibco, Carlsbad, CA, USA), and penicillin/streptomycin (Gibco, Carlsbad, CA, USA). The cultures were further split at 1:5–1:6 ratio by disaggregation with Accutase when the cells reached approximately 80% confluence.

### 2.3. Virus

CeHV-2 was a kind gift by David Brown and Matthew Jones, Public Health England. Sequencing confirmed its identity with strain B264 [8,28]. The virus was propagated on Vero76 cells by infection at MOI 0.01 and harvested once complete cytopathic effect had developed.

### 2.4. Fosmid Cloning of CeHV-2 DNA

For cloning of CeHV-2 DNA into fosmid vector DNA was prepared from virus particles as follows. CeHV-2 virions from up to 18 mL cell culture supernatant (cells infected for 2–3 days) were concentrated by ultracentrifugation (Thermo WX Ultra 80; Surespin rotor, 28.000 rpm, 70 min). The virion pellet was resuspended in 100 μL PCR lysis buffer (50 mM KCl, 10 mM Tris pH8.3, 1.5 mM MgCl_2_, 0.001% gelatine, 0.5% triton X-100, 35 μg/mL Proteinase K) and incubated for 1 h at 56 °C. Then the DNA was sheared 5 times through a 27G needle and precipitated after addition of 40 μL 5 M NaCl and 125 μL of isopropanol. After washing twice in 70% ethanol the DNA pellet was dried, dissolved in 10 μL H_2_O. Afterwards the DNA was processed for fosmid cloning as recommended by the manufacturer (CopyControl Fosmid Library Production Kit; Lucigen, Middleton, WI, USA). Briefly, the DNA was treated with an End-Repair Enzyme Mix and then separated on a 0.8% low melting point agarose using control fosmid and DNA size marker (GeneRuler High Range DNA Ladder, Thermo Fisher, Waltham, MA, USA) in the neighboring lanes as reference. After staining the reference lanes with ethidium bromide, a gel slice encompassing about 20–40 kb fragments of the CeHV-2 DNA was cut out, avoiding exposure of this DNA to ethidium bromide or UV light. The gel slice was melted at 70 °C for 15 min and incubated overnight at 45 °C in the presence of GELase. After precipitation of the DNA, as recommended by the manufacturer, the DNA pellet was dissolved in water and 250 ng was used for ligation to 500 ng Eco72I-linearized pCC1FOS vector. The ligation mixture was then packaged into lambda particles using MaxPlax Packaging Extracts, which were used for transduction of E. coli EPI300 cells, according to the protocols supplied by the manufacturer.

### 2.5. Characterization of Fosmids

**Figure 1:**
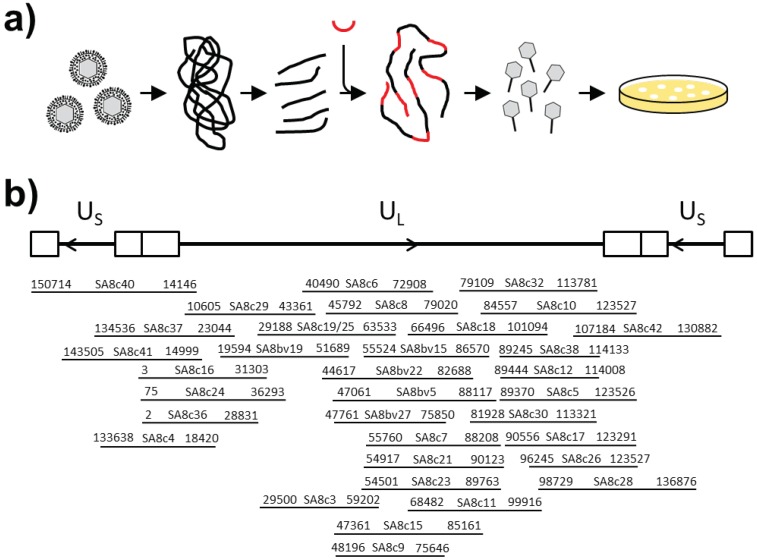
Cloning and characterization of CeHV-2 fosmids. (**a**) Scheme of CeHV-2 genome cloning. Viral DNA was isolated from virus particles, sheared, end-repaired and size fractionated on an agarose gel. Fractionated fragments were ligated into fosmid vector pCC1FOS (red) and packaged into phage lambda particles, which were then used to transduce cells of E. coli strain EPI300. Individual colonies were further characterized by colony PCR, end-sequencing, and restriction digest. (**b**) An overview of all fosmid clones that are characterized by colony PCR and end-sequencing. The genome of CeHV-2 is schematically represented on top, with U_S_ regions drawn on both sides of the U_L_ region. The direction of gene order, in the U_L_ and U_S_ regions, is indicated by arrows and inverted repeats are drawn as boxes. The positions of fosmid inserts are drawn as lines. The names of the fosmids and nucleotide positions of the insertions are given above the respective lines.

Colonies containing fosmids were first screened with PCR primers positioned every 15 kbp of the CeHV-2 genome (NC_006560) [8], to gain information about the part of genome present. The start and end of the fragment was determined by sequencing for selected clones (Figure 1b) and the integrity of the insert was confirmed by restriction digest. A set of five clones with overlapping ends that were able to successfully rescue the virus was finally analyzed by next generation sequencing.

### 2.6. Rescue of CeHV-2

For virus rescue, Vero 76 cells were seeded in 12 well plates at 10^5^ cells per well. Fosmid DNAs were linearized by digestion with HindIII, ethanol precipitated and dissolved in sterile water. A mixture of fosmids covering the whole genome (1 μg for each fosmid) was transfected using Lipofectamine 2000 (Thermo Fisher, Waltham, MA, USA) according to the protocols of the manufacturer. Usually, cytopathic effects (cell rounding, syncytia) were visible after 2–3 days and the virus was harvested once all cells showed signs of infection.

### 2.7. Replication Kinetics and Plaque Assay

To measure virus replication, Vero76 cells were seeded in 24 well plates at 5 × 10^4^ cells per well. On the next day, the cells were infected in triplicate with a cell-free virus at MOI 1. After 1 h incubation, the cells were washed with PBS and incubated in 1 mL fresh medium until harvest. To measure the cell-free virus, the supernatant was transferred into Eppendorf tubes, centrifuged 5 min at 4000 rpm to remove cellular material and frozen at −80 °C. To determine the titers of cell associated virus, cells were detached with accutase, transferred to Eppendorf tubes, and collected by centrifugation. After resuspension in 1 mL medium, cells were subjected to three freeze-thaw cycles and cleared from cellular material by centrifugation for 5 min at 4000 rpm. The supernatant was frozen at −80 °C.

Virus titers were determined by plaque assay. For this, Vero76 cells were seeded in 24 well plates at 9 × 10^4^ cells per well. On the next day, cells were infected with serial 10-fold dilutions of harvested supernatants for 1 h at 37 °C. After removal of virus inoculum, 0.5 mL overlay medium containing avicel (FMC, Philadelphia, PA, USA) [29] was added and cells incubated for 3–4 days. For harvest, avicel overlay medium was removed and the cells washed in PBS to remove the remains of avicel. Then, the cells were fixed with cold methanol for 10 min at −20 °C and dried after removal of methanol. Fixated cells were stained with crystal violet solution (0.2% crystal violet, 20% ethanol, 3.5% formaldehyde) for 2 min at room temperature and washed twice with water. Plaques were counted and virus titer calculated as plaque forming units (pfu) per mL.

### 2.8. Recombineering

For recombineering by en passant mutagenesis [19], fosmids were transferred into E. coli GS1783 [18], which expressed phage recombinases after heat induction and I-SceI under arabinose control. Plasmids pcDNA3-EGFP-en, pHW2000GG-seg8-A/PR/8/34-2A-Gluc-en, pHW2000GG-seg8-A/PR/8/34-M2A-mCherry-en, pcDNA3-iRFP670-en were used as template in PCR reaction employing primer pairs ep-SA8-ICP4-EGFP-5/ep-SA8-ICP4-EGFP-3, ep-SA8-ICP4-Gluc-3/ep-SA8-ICP4-2A-5, ep-SA8-UL35-EGFP-5/ep-SA8-UL35-EGFP-3 and ep-SA8-UL10C-R670-5/ep-SA8-UL10C-R670-3, respectively. The resulting PCR products contained the en passant reporter gene cassette, flanked by 50 bp sequence ends homologous to the targeted gene. In all cases, reporter genes were targeted towards the C-termini of the ICP4, UL35 or UL10 coding regions. PCR products were treated with DpnI to inactivate template plasmids and gel purified.

E. coli GS1783 strains harboring the respective fosmids (SA8c28 and SA8c41 for ICP4; SA8c6 and SA8c18 for UL35; SA8c29 for UL10) were grown in LB-medium in presence of 25 μg/mL chloramphenicol at 30 °C until the culture reached an optical density (600 nm) of 0.5–0.7. Then, the culture flasks were transferred to a water bath shaker and incubated at 42 °C for 15 min, to induce recombinase enzymes. After cooling in an ice water bath for 20 min, the cells were collected by centrifugation and washed three times in cold sterile water. The pellet was finally resuspended in an equal volume of 10% glycerol. For electroporation 100 μL cells were mixed with PCR product and transferred to a 2 mm electroporation cuvette. Electroporation was performed at 2500 V, 25 μF and 200 Ω in a Biorad Gene Pulser. The cells were immediately transferred to 1 mL LB medium, incubated for 2 h at 30 °C and plated on LB agar plates containing 25 μg/mL chloramphenicol and kanamycin. After incubation for 2 days at 30 °C, the colonies were analyzed by colony PCR employing primers positioned outside the respective targeted regions. To remove the kanamycin gene and restore the respective reporter genes, positive colonies were grown over night at 30 °C in LB medium containing 25 μg/mL chloramphenicol and kanamycin. Then 2 mL LB medium containing 25 μg/mL chloramphenicol were inoculated with 100 μL overnight culture incubated for 3 h at 30 °C. To induce I-SceI enzyme in E. coli GS1783 strains 2 mL LB medium containing 25 μg/mL chloramphenicol and 1% L-arabinose were added to the culture and incubated for 1 h at 30 °C. Afterwards the culture was transferred to a water bath shaker at 42 °C for 30 min, followed by incubation at 30 °C at 1–2 h. The cells were then diluted and 100 μL of 10^−3^ und 10^−4^ dilutions were plated on LB agar plates containing 25 μg/mL chloramphenicol and 1% L-arabinose. The colonies harboring the reporter genes without kanamycin marker were identified by colony PCR. PCR amplificates were sequenced to confirm the sequence of the desired clones and fosmids were characterized for integrity by restriction digest.

### 2.9. Infection and Luciferase Assay

For single cycle infection assays, the cells were seeded in 96 well plates at 10^4^ cells/well. On the next day, medium was removed and cells infected with SA-ICP4-2A-Gluc virus in a volume of 100 μL with MOI1, 0.1 or 0.01 for 1 h. Subsequently, the cells were washed four times with PBS followed by addition of 150 μL fresh medium. For luciferase measurement 25 μL supernatant was used. All samples were processed as 8-fold replicates.

For multi cycle experiments, cells were seeded in 6-well plates at 2.5 × 10^5^ cells/well. On the next day cells were infected with 100 pfu of SA-ICP4-2A-Gluc virus in a volume of 1 mL for 1 h. Infectious supernatant was then replaced by 2 mL fresh medium. For inhibition of viral replication (Glentham Life Sciences Ltd, Corsham, UK), infection and subsequent cultivation was carried out in the presence of acyclovir (8 and 32 μg/mL). At time intervals of 6–18 h 25 μL, the aliquots were collected and stored at −20 °C. After five days of collecting, luciferase activity was determined for all samples. For multi cycle experiments triplicates of each cell line were measured. Luciferase activities were measured in a Plate Chameleon V (Hidex, Turku, Finland) instrument. As substrate, coelenterazine (PJK, Kleinblittersdorf, Germany) was diluted in D-PBS (with Ca and Mg) to a final concentration of 1.5 mM. We usually measured the background values around 3.000 cps (aliquots of supernatant taken before infection).

### 2.10. Next-Generation Sequencing and Assembly

Library preparation of fosmid-DNA was performed using the NEBnext Ultra II FS DNA Library Prep Kit (New England BioLabs, Ipswich, MA, USA) following the manufacturer’s protocol for <100 ng DNA input (30–46 ng input amount). The quality was controlled using an Agilent Bioanalyzer (Agilent Technologies, Santa Clara, CA, USA) and the libraries were subsequently sequenced on an Illumina MiSeq (Illumina, San Diego, CA, USA) with a 600v3 sequencing kit (2 × 300 bp paired-end reads). The sequence data were trimmed and quality controlled using fastp [30], and de novo assembled in the CLC Genomics Workbench (version 10; QIAGEN, Hilden, Germany). Sequence annotations were transferred from the reference vector constructs using Geneious Prime (Biomatters, Auckland, New Zealand). The CeHV-2 sequence coverage was above 99% for all sequenced fosmids and sequences were identical to the reference genome (Genbank NC_006560).

## 3. Results

### 3.1. Cloning and Rescue of CeHV-2

To establish a recombinant system for CeHV-2, we first fragmented the viral genome and generated a set of fosmid clones with overlapping ends of at least 1 kb. For this, viral DNA was isolated from virions, sheared to fragments of 30–40 kb, and size fractionated on a low percentage agarose gel (Figure 1a). The recovered fragments were ligated into fosmid vector pCC1FOS and packaged into phage lambda particles for transduction of E. coli cells.

Individual E. coli colonies were then characterized by colony PCR with primer pairs that were positioned every 15 kbp to map the position of the cloned fragments in the viral genome. The 5′ and 3′ ends of selected clones were then sequenced to determine the exact positions of the inserts in the viral genome (Figure 1b), and further characterized by restriction digest to check for integrity of the inserts.

**Figure 2:**
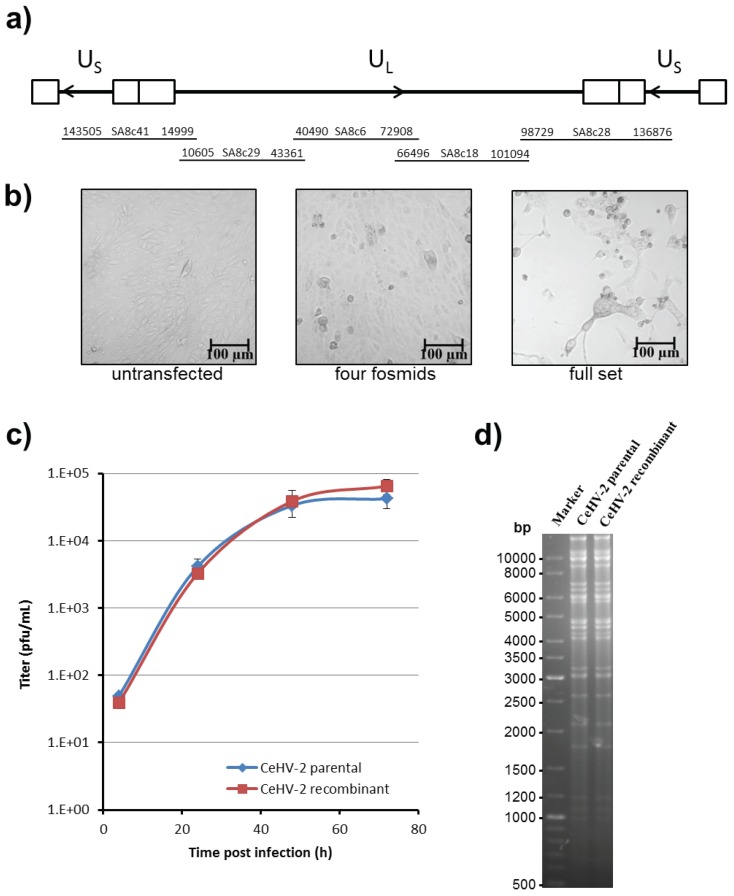
Rescue and characterization of recombinant CeHV-2 virus. (**a**) Schematic depiction of the CeHV-2 genome as in Figure 1b along with the fosmid clones used for rescue. (**b**) Cells were transfected with a linearized set of the fosmids. Brightfield images of untransfected Vero76 cells (left panel) and Vero76 cells transfected with a partial (four fosmids; fosmid SA8c6 missing; middle panel) and a full set (right panel) taken 3 days after transfection are shown below. Scale bars represent 100 μm. (**c**) Replication kinetics of parental and recombinant CeHV-2 viruses on Vero76 cells infected with MOI 1. Culture supernatant was harvested at the indicated time points and virus titer determined by plaque assay. The results of a representative experiment, carried out with triplicate samples, are shown. Similar results were obtained in a separate experiment. Error bars indicate standard deviation (SD). (**d**) Restriction digest with BamHI of genomes of parental and recombinant CeHV-2 viruses isolated from virus particles.

A set of five fosmid clones with ends overlapping between 2.3–6.4 kbp was chosen for the rescue of infectious CeHV-2 (Figure 2a). The fosmid clones were positioned on the genome in such a way that both copies of the inverted repeats were located on separate fosmids. The fosmid DNAs were first linearized at a unique HindIII site present in the multiple cloning site of pCC1FOS and subsequently transfected into Vero76 cells. At 3 days after transfection, clear cytopathic effects were visible, as demonstrated by rounded cells and formation of syncytia (Figure 2b, right panel). While, no changes in the cell monolayer were seen with untransfected cells (Figure 2b, left panel). If one fosmid was excluded during transfection, numerous rounded cells were observed but no formation of syncytia or plaques was detected (Figure 2b, middle panel). The recovered virus could be passaged to fresh uninfected Vero76 cells, and similar results were obtained for a second set of fosmids with overlapping regions shifted relative to the first set (Supplementary Information Figure S1).

To further characterize the recovered virus, we compared the replication of the recombinant virus with the parental virus. For this, Vero76 cells were infected at MOI 1 with both viruses and culture supernatants were collected. As shown in Figure 2c, both parental and recombinant CeHV-2 viruses exhibited almost identical replication kinetics. Finally, we compared the restriction pattern of viral DNA, prepared from virions of parental and recombinant viruses and found that they were identical (Figure 2d). Thus, we could demonstrate the rescue of a recombinant CeHV-2 virus from fosmids, and we could show that wt and recombinant viruses replicated with comparable efficiency.

### 3.2. Recombineering of CeHV-2

**Figure 3:**
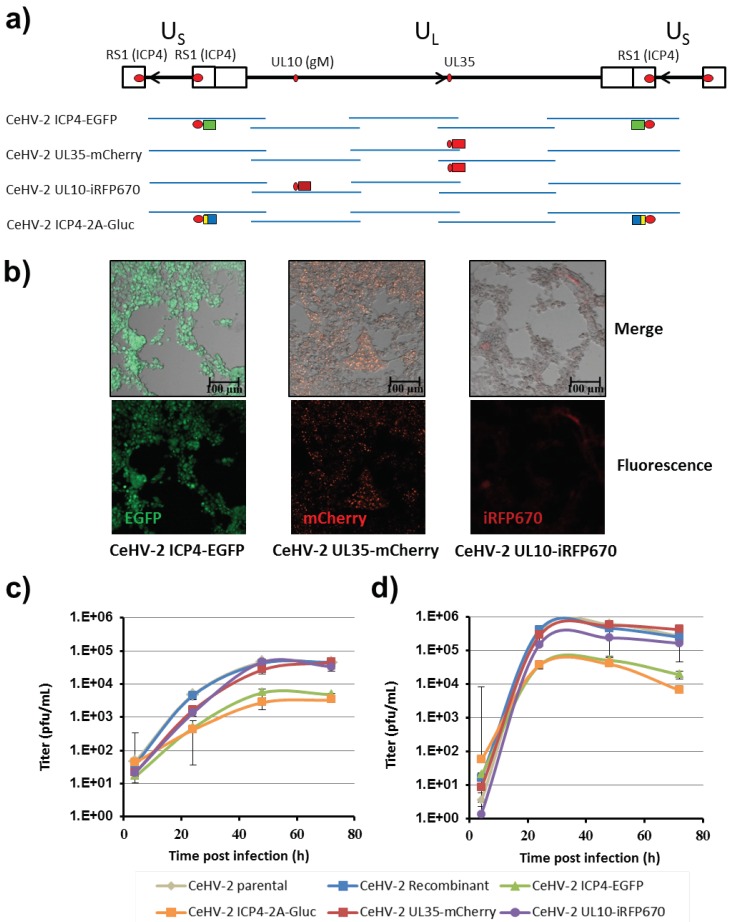
Generation and characterization of CeHV-2 reporter viruses. (**a**) Overview of modifications introduced into CeHV-2 fosmids. The genome of CeHV-2 is drawn, as in Figure 1b. The genes that were modified are indicated as red circles. Reporter genes coding for EGFP (green boxes), mCherry or iRFP670 (bright and dark red boxes) or 2A-Gluc (yellow and blue boxes) were inserted into the respective fosmids. In all cases, the reporter proteins were fused to the C-termini of the viral proteins. The names of the resulting CeHV-2 reporter viruses are indicated on the left side. (**b**) Rescue of CeHV-2 fluorescent reporter viruses. Vero76 cells were transfected with the wt and modified fosmids shown in (a) to generate CeHV-2 ICP4-EGFP (left), CeHV-2 UL35-mCherry (middle) or CeHV-2 UL10-iRFP670 (right). Plaque formation was monitored 6 d after transfection by confocal microscopy. Images show the respective fluorescent signal alone (fluorescence; lower panels) or in an overlay with a brightfield image (merge; upper panels). Scale bars represent 100 μm. (**c**,**d**) Replication kinetics of parental and recombinant CeHV-2 viruses on Vero76 cells infected at MOI 1. Culture supernatant or cells were harvested at the indicated time points. Cells were subjected to three freeze-thaw cycles to release cell-associated virus. Titers were determined by plaque assay for viruses released into the supernatant (**c**) or for cell-associated viruses (**d**). The results of representative experiments, carried out with triplicate samples, are shown in panels c and d, and were confirmed in two separate experiments. Error bars indicate SD.

We next inserted reporter genes into the fosmids, in order to easily detect replication of the recombinant viruses (Figure 3a). For this, we used recombineering and targeted three genes: First, RS1, which encodes the immediate-early protein ICP4. The RS1 gene is located in the inverted repeats of the US region and thus two copies of the gene are present in the viral genome. Since both copies were present on different fosmids, we were able to manipulate both copies independently and in parallel. Second, UL35, a late gene encoding the small capsid protein. Third, UL10, a late gene encoding glycoprotein M. Since UL35 was present in the overlapping region of two fosmids, it was necessary to modify both fosmids. We chose fluorescent proteins EGFP, mCherry and iRFP670 as reporter genes and fused them to the C-termini of ICP4, UL35, and UL10, respectively, with a Gly-Ser-Gly linker separating reporter and viral proteins (Figure 3a). In addition, we fused Gaussia luciferase (Gluc) to the C-terminus of ICP4 and inserted a Gly-Ser-Gly linker and a PTV1-derived 2A stop-go sequence between the two proteins. This should enable secretion of Gluc and allow for the sensitive measurement of infection. All alterations were introduced by en passant mutagenesis and controlled by PCR and sequencing of the modified regions.

To rescue the viruses expressing the modified proteins, the respective linearized engineered fosmids were co-transfected with linearized wt fosmids necessary to cover the SA genome (Figure 3a). Fluorescent plaques demonstrating successful rescue were clearly visible after 3 days for all recombinant viruses bearing fluorescent proteins, and covered large areas of the cell monolayer at 6 days post transfection (Figure 3b). However, plaques generated by CeHV-2 ICP4-EGFP were smaller in size than plaques formed by CeHV-2 UL35-mCherry or CeHV-2 UL10-iRFP670. Apart from the viruses carrying fluorescent reporters, the CeHV-2 ICP4-2A-Gluc virus was also successfully rescued, as judged by development of CPE (not shown) and release of Gluc (see below).

We next characterized the replication of the recombinant reporter viruses and compared it with that of wt parental and wt recombinant viruses. As shown in Figure 3c, virus titers in the supernatant increased up to 2 dpi and then reached a plateau. CeHV-2 UL35-mCherry and CeHV-2 UL10-iRFP670 grew to titers similar to parental or recombinant CeHV-2, while for both viruses with modified ICP4, CeHV-2 ICP4-EGFP and CeHV-2 ICP4-2A-Gluc, the titers were about one log lower (Figure 3c), indicating a moderate replication deficiency. When the titers of cell-associated viruses were compared, they were found to be generally higher and the plateau was reached after 1 dpi (Figure 3d). As in the supernatant, the titers of CeHV-2 ICP4-EGFP and CeHV-2 ICP4-2A-Gluc were about one log lower than for the other viruses. Finally, we asked whether the reduced replication of the viruses encoding ICP4 fused to reporter proteins was due to reduced ICP4 expression. Indeed, ICP4 levels in wt CeHV-2 infected cells were higher than those detected in CeHV-2 ICP4-2A-Gluc and CeHV-2 ICP4-EGFP infected cells [31], suggesting that the reduced replication of the reporter viruses might be due to reduced ICP4 expression.

In summary, we could demonstrate fosmid modification by recombineering and rescue of several viruses carrying reporter genes fused to viral proteins. These viruses grew to high titers, albeit viruses with modification of ICP4 achieved somewhat reduced titers.

### 3.3. Tropism of CeHV-2 for Different Cell Lines

**Figure 4:**
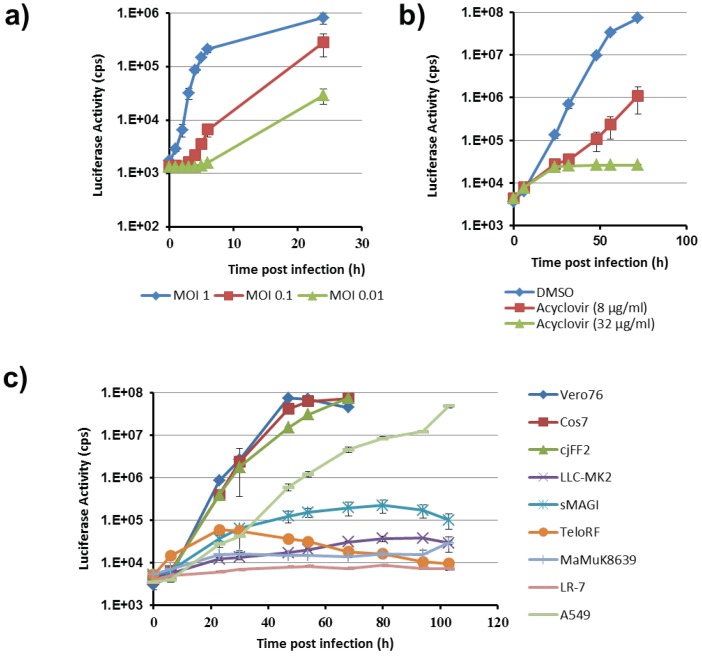
Kinetics of luciferase activity of CeHV-2 ICP4-2A-Gluc in infected cells. (**a**) Vero76 cells were infected at the indicated MOIs with CeHV-2 ICP4-2A-Gluc. Cell culture supernatant was harvested at the indicated time points after infection and luciferase activity determined. The results of a single, representative experiment are shown and were confirmed in a separate experiment. Standard deviation is given for octuplicate samples. (**b**). Multicycle replication kinetics in Vero76 cells infected with low virus dose (100 pfu; MOI 0.0004). Infection and cultivation was done in the medium containing acyclovir or solvent (DMSO). Aliquots of cell culture supernatant were continuously harvested over 3 days followed by measurement of luciferase activity. The results of a single, representative experiment carried out with triplicate samples are shown and were confirmed in a separate experiment. Error bars indicate SD. (**c**) Multicycle replication kinetics on cell lines derived from different species. Cells were infected with low virus dose (100 pfu; MOI 0.0004) and aliquots of cell culture supernatant continuously harvested over 5 days followed by measurement of luciferase activity. The results of a single experiment carried out with triplicate samples are shown and are representative of a total of 2–4 separate experiments. Error bars indicate standard deviation (SD).

We next employed our reporter viruses to examine the cell line tropism of CeHV-2. For this, we concentrated on CeHV-2 ICP4-2A-Gluc, since Gaussia luciferase (Gluc) allows for highly sensitive detection and, being secreted into the supernatant, continuous sampling. We first analyzed the kinetics of Gluc release from Vero76 cells infected at different MOIs. Upon infection at MOI 1, we observed an increase of Gluc activity in the culture supernatant at 1-2 hpi, which started to level off at 5–6 hpi (Figure 4a). For lower MOIs the rise in Gluc activity started later but for both MOIs a rise of more than 10-fold (MOI 0.01) and 100-fold (MOI 0.1) above background was detected at 24 hpi. Moreover, acyclovir, which impedes viral genome replication, but not immediate early gene expression, reduced Gluc activity close to background, demonstrating that robust reporter activity required viral replication (Figure 4b). Thus, the reporter activity upon CeHV-2 ICP4-2A-Gluc infection is dependent on MOI and viral replication and the reporter virus should be suitable to detect even low levels of infection.

We next tested the ability of CeHV-2 to infect different cell lines. For this, cell lines of human, non-human primate (NHP) and mouse origin were infected at MOI 0.0004 and Gluc levels in culture supernatants were measured for five days, a time frame that allows multi-cycle viral replication. As shown in Figure 4c, CeHV-2 replication was most robust in Vero76 and Cos7 cells, both derived from African green monkey, the suspected natural host of CeHV-2. Similar replication was seen in fibroblasts from marmosets (*Callithrix jacchus*). The human lung cell line A549 also supported robust viral replication, while no replication was detected in mouse LR-7 cells (Figure 4c). Finally, all four rhesus macaque-derived cell lines (LLC-MK2, MaMuK8639, both kidney; sMAGI, mammary tumor; TeloRF, fibroblast) were barely susceptible to CeHV-2 replication. Collectively, these results show that CeHV-2 can replicate in diverse cell lines of human and NHP origin but also indicate certain previously undetected limitations in cell type and/or species tropism of CeHV-2.

## 4. Discussion

Here, we describe a fosmid-based system for the generation of recombinant CeHV-2. This system allowed the rescue of recombinant CeHV-2 that replicated with the same efficiency as uncloned wt virus. Moreover, we successfully equipped CeHV-2 with reporter genes that allowed for convenient quantification of viral replication. Finally, we found that CeHV-2 infected human, African green monkey and marmoset derived cell lines, but failed to infect several rhesus macaque derived cell line. The fosmid-based system described should be suitable for the rescue of diverse herpesviruses, and CeHV-2 plasmids should be helpful in future studies of the biological properties of this virus.

The fosmid approach chosen here combines advantages of cosmids and BACs. BACs are now state-of-the art for modification of herpesviral genomes by recombineering. However, it has been difficult to seamlessly alter diploid genes present in inverted repeat regions of many herpesviral genomes [32]. In alphaherpesviruses these regions are of special interest, as they contain important genes for lytic and latent regulation. In cosmid-based systems, diploid genes could be modified separately [10,11]. Moreover, cosmids offer a high level of biosafety, since only parts of the genome are handled at a time. However, cosmids, are present at medium to high copy-numbers in bacterial cells and have proven difficult to modify using conventional or recombination-based protocols [11,12,33]. In contrast, fosmids are only present at low copies, since they contain a F-factor-derived low copy replication origin for plasmid maintenance and a separate inducible origin for DNA production [21], and the present study confirms that they are amenable to modification by recombineering [22,23,24,25]. Thus, the approach employed here for generation and modification of CeHV-2 should also be suitable for other alphaherpesviruses.

The separation of diploid regions of the genome on separate fosmids allowed the parallel and seamless alteration of a diploid gene (RS1/ICP4), thus minimizing the number of consecutively required steps. In this way, we could recover reporter viruses expressing ICP4-EGFP fusion proteins or co-expressing ICP4-2A and Gluc. In addition, reporter genes could be introduced into other regions of the CeHV-2 genome and replication competent viruses were obtained. If viral open reading frames, that are located in the overlap between two fosmids, are to be modified, as UL35 in our case, both copies of the gene need to be modified. The availability of several reporter constructs may allow the rescue of multi-colored viruses, and we recently rescued a CeHV-2 ICP4-EGFP UL35-mCherry virus [34] However, the generation of viruses encoding three reporter genes failed so far and the underlying reasons remain to be investigated. In summary, we applied recombineering to generate CeHV-2 reporter viruses, indicating that our system is suitable to genetically modifying large recombinant DNA viruses, such as herpesviruses.

The Gluc-expressing reporter virus allowed us to analyze cell line tropism of CeHV-2 and inhibition experiments with acyclovir confirmed that the reporter activity, observed in these experiments, reflected viral replication. The virus replicated efficiently in Vero76 and Cos7 cell lines from African green monkey (*Cercocebus* spp.). This is in agreement with previous reports on replication of CeHV-2 in Vero cells [8,9]. In addition, we observed robust replication in marmoset fibroblasts, which has not been reported so far. Replication in human A549, Huh7.5, HeLa and HEK293T (not shown) was also detected, in agreement with reports on CeHV-2 replication in human foreskin fibroblasts [9], KB (human epithelial carcinoma) or fetal diploid lung cells [35]. In contrast, no appreciable replication was detected in cell lines derived from rhesus macaques (LLC-MK2, sMAGI, TeloRF, MaMuK8639). This is in contrast to a previous report [35] showing productive CeHV-2 infection of the rhesus macaque cell line LLC-MK2. We can, at present, not rule out that these differences are due to use of reporter virus in the present and wt virus in the previous study. However, our preliminary data showed that infection of LLC-MK2 cells with wt virus was very inefficient (not shown). Thus, we cannot dismiss the possibility that CeHV-2 may generally be able to infect rhesus macaque cells and that the cell lines tested here might lack host factors required for viral replication or that it might express antiviral factors that suppress CeHV-2 infection.

In summary, we report fosmid-based cloning of a herpesvirus genome as an alternative approach to generating recombinant viruses, and we demonstrate that CeHV-2 reporter viruses generated by recombineering allow the investigation of CeHV-2 infection.

## Supplementary Materials

The following are available online at https://www.mdpi.com/1999-4915/11/11/1026/s1, Figure S1: Rescue of recombinant CeHV-2 virus.
