# Supplementary material for: A Fosmid-Based System for the Generation of Recombinant Cercopithecine Alphaherpesvirus 2 Encoding Reporter Genes
Source: Viruses. 2019 Nov 5;11(11):1026. doi: 10.3390/v11111026 (PMC6893520; doi:10.3390/v11111026)
Supplement: Supplementary file 1 [file viruses-11-01026-s001.pdf]

Supplementary Information Figure S1 — Chukhno *et al.*

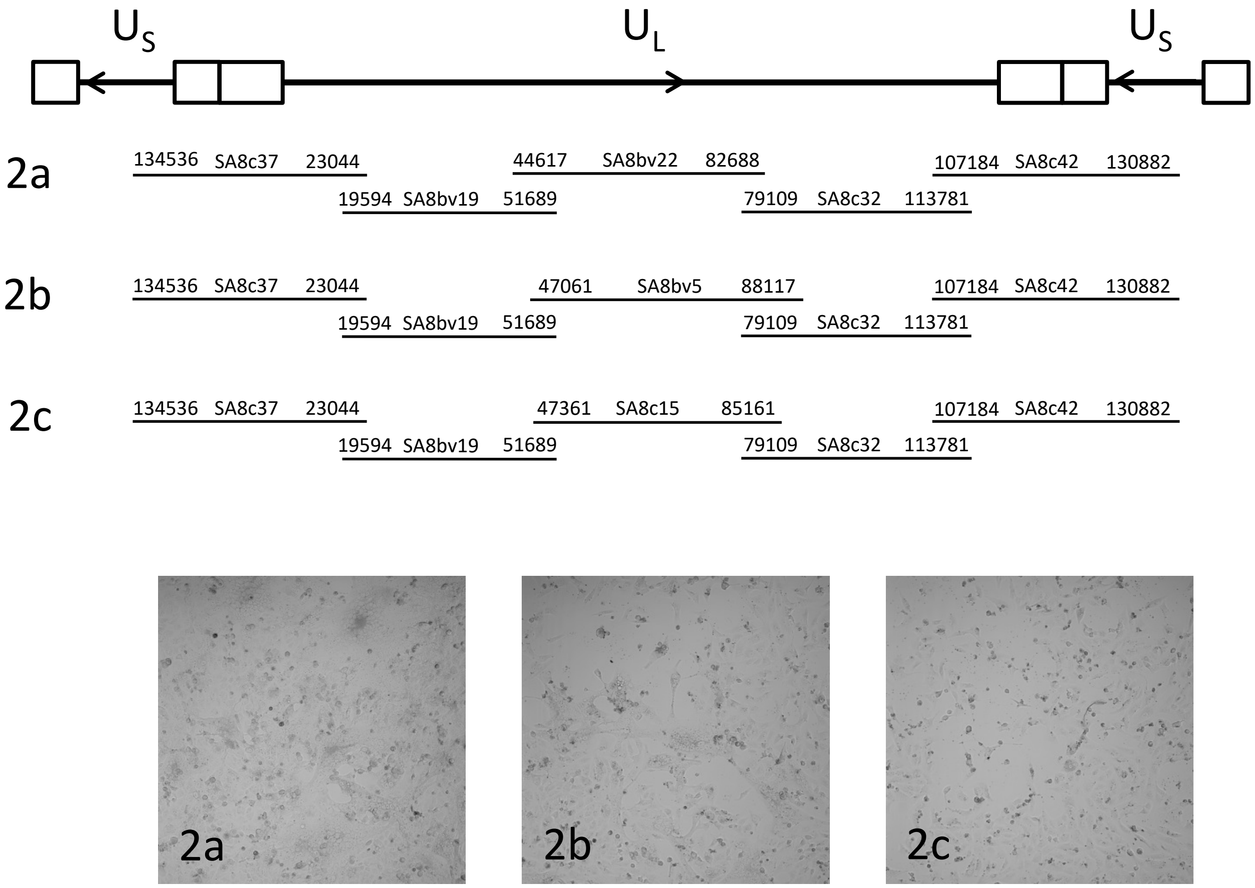

Figure S1. Rescue of recombinant CeHV-2 virus. Schematic depiction of the CeHV-2 genome along with the fosmid clones used for rescue as in figure 1b and 2a. Cells were transfected with linearized sets of the fosmids. The three sets 2a, 2b and 2c differ in the central fosmid. Brightfield images of transfected Vero76 cells taken 2 days after transfection are shown below.
